# Effects of different intervention combined with resistance training on musculoskeletal health in older male adults with sarcopenia: A systematic review

**DOI:** 10.3389/fpubh.2022.1037464

**Published:** 2023-01-06

**Authors:** María del Carmen Carcelén-Fraile, María Florencia Lorenzo-Nocino, Diego Fernando Afanador-Restrepo, Carlos Rodríguez-López, Agustín Aibar-Almazán, Fidel Hita-Contreras, Alexander Achalandabaso-Ochoa, Yolanda Castellote-Caballero

**Affiliations:** ^1^Department of Health Sciences, Faculty of Health Sciences, University of Jaén, Jaén, Spain; ^2^ZIPATEFI Research Group, Faculty of Health Sciences and Sports, University Foundation of the Área Andina, Pereira, Colombia; ^3^GIP Pedagogy Research Group, Faculty of Distance and Virtual Education, Antonio José Camacho University Institution, Santiago de Cali, Colombia; ^4^Clinical Director at Sinapse Neurology, CEO Mbody Research and Formation Group, University Schools Gimbernat, Attached to the University of Cantabria, A Coruña, Spain

**Keywords:** sarcopenia, resistance training, older adults, men, protein supplementation, aerobic training, nutritional supplementation

## Abstract

**Objectives:**

Nowadays, there is a significant increase in the elderly population in many countries around the world, and sarcopenia is one of the most common consequences of this with resistance training being one of the best treatments. Hence, this systematic review was conducted to determine what are the effects of different combinations of resistance training-based interventions on the musculoskeletal health of older male adults with sarcopenia

**Methods:**

This systematic review was performed following the PRISMA 2020 guidelines. The search was performed between February and August 2022 in three electronic databases: Pubmed (MEDLINE), Web of Science (WOS) and Scopus employing different keywords combined with Boolean operators. Only 13 articles were included out of the initial 1,019.

**Results:**

The articles studied the effects of resistance training combined with other interventions, 6 articles combined it with protein and vitamin supplementation, 4 with protein supplements only, while 3 combined it with aerobic training, finding beneficial results mainly on strength, functionality, and body composition.

**Conclusion:**

Resistance Training combined with Aerobic Training or nutritional supplements has better effects than Resistance Training alone in older male adults with sarcopenia.

**Systematic review registration:**

https://www.crd.york.ac.uk/prospero/#recordDetails, identifier: CRD42022354184.

## 1. Introduction

Currently, there is evidence of a significant increase in the elderly population in many countries of the world, including Spain. As a result, more and more people are becoming susceptible to the diseases associated with aging (1). According to the WHO, the percentage of people over 60 years of age is increasing worldwide, reaching 1 billion in 2019 and it is expected that by 2050 this number will increase to 2.1 billion (2). This has attracted the attention of multiple researchers, becoming one of the most relevant topics today, even considering aging as a global public health issue (3). Understanding the processes of aging will allow the development of new and better interventions that can alleviate the adverse effects that time brings on human beings.

Sarcopenia is one of the consequences of aging, consisting of a progressive loss of muscle mass with a consequent loss of strength, accompanied by a decrease in physical performance (4). In 2010 The European Working Group on Sarcopenia in Older People (EWGSOP) established a definition updated in 2018, which recognized sarcopenia as a muscular pathology characterized by the gradual loss of both, muscle mass and strength affecting the quality of life, with risk of disability and even death (5, 6). Additionally, since 2016 it is considered by the WHO as a disease (7). This pathology is present in 6–22% of people over 65 years of age, being this percentage higher when the subjects belong to home residences (14–38%), are hospitalized (10%) (7) or are over 80 years of age (8). Sarcopenia, in the same way as aging, has been considered a public health problema (9), with a higher prevalence in men than in women (11 vs. 2%) when the EWGSOP definition is used (10).

This disease has been associated with an increased risk of mortality, risk of falls, fractures and a marked decrease in physical capacity (11). Occasionally, it often occurs in association with nervous depression, mainly in men, where a direct relationship between nervous depression and muscle mass loss has been observed (12). To date, there is no pharmacological treatment that has been approved for use in humans; however, animal research in this field is promising (13). On the other hand, the implementation of non-pharmacological interventions based on exercise and nutritional interventions have shown favorable results (14).

Resistance training or strength training is one of the options for exercise-based interventions, in which the subject uses his or her body and strength to oppose an external resistance (resistance machines, free weights, elastic bands, etc.) or internal resistance (body weight) (15). Among the multiple effects of exercise in people with sarcopenia is the increase in strength and muscle mass, which improves the functionality of the individual, in addition to combating oxidative stress caused by the aging process (16–18).

During the last few years, a large amount of evidence and articles on physical exercise and its effects on sarcopenia have been published (19–21), however, these have mainly focused on the female population and have rarely considered complementary interventions that enhance the effects of exercise. There is a great need to establish a cost-effective treatment to mitigate the effects of sarcopenia, especially in men, who have a higher prevalence of this disease. For this reason, this study aims to determine what are the effects of different combinations of resistance training-based interventions on the musculoskeletal health of older male adults with sarcopenia.

## 2. Materials and methods

This systematic review was performed following the PRISMA 2020 guidelines (22). The protocol followed in this review was registered in the International Prospective Register of Systematic Reviews PROSPERO under code CRD42022354184.

### 2.1. Eligibility criteria

Articles were selected based on the following inclusion criteria: (i) studies that evaluated the effects of resistance training in older male adults diagnosed with sarcopenia. (ii) studies in which at least one of the groups studied had received an intervention based on resistance training combined with another type of intervention and that evaluated a variable related to muscle health. Gray literature was not considered, and no filter associated with date of publication, publishing country or language were applied.

### 2.2. Information sources

The search was performed between February and August 2022 in three electronic databases: Pubmed (MEDLINE), Web of Science (WOS) and Scopus. A snowball search was not performed to avoid non-reproducibility of the protocol used.

### 2.3. Search strategy

The search strategy was designed specifically for this study employing keywords combined with Boolean operators, resulting in the following search string: (“older adults” OR “older men” OR “elderly” OR “seniors” OR “aging” OR “elderly people” OR “Male older adult”) AND (“Resistance training” OR “Endurance training”) AND (“Musculoskeletal health” OR “strength” OR “Skeletal muscle index” OR “SKI” OR “muscular quality” OR “Bone mineral density” OR “BMD”) AND (“Sarcopenia”).

### 2.4. Selection process

The entire article selection process was carried out using the Rayyan QCRI application (23) (https://rayyan.qcri.org/welcome, accessed on 25 February 2022). Initially, two of the authors (M.d.C.C.-F. and A.A.-A.) were in charge of eliminating all the duplicate articles and then reviewing the titles and abstracts, choosing the articles that would be read in full text. Finally, two different authors (D.F.A.-R. and F.H.-C.) read the full text of the articles that met the eligibility criteria and blindly gave their verdict. When disagreements arose, a third author was in charge of solving them.

### 2.5. Data extraction

The main variable of this study was musculoskeletal health measured through strength, the skeletal muscle index, muscular quality, or the bone mineral density. Additionally, the authors, year and country of publication, the type of additional intervention employed, and the characteristics of resistance training were retrieved.

### 2.6. Assessment of methodological quality

The methodological quality of the selected articles was measured using the PEDro scale (24). This instrument consists of 11 items that can be answered as YES (1 point) or NO (0 points), the maximum score is 10 points since the first item is not used in the final score calculation. A score between 0 and 3 was considered “Poor” quality; between 4–5 “Fair”, 6–8 “Good” and >9 “Excellent” (25).

## 3. Results

### 3.1. Selection of the studies

**Figure 1:**
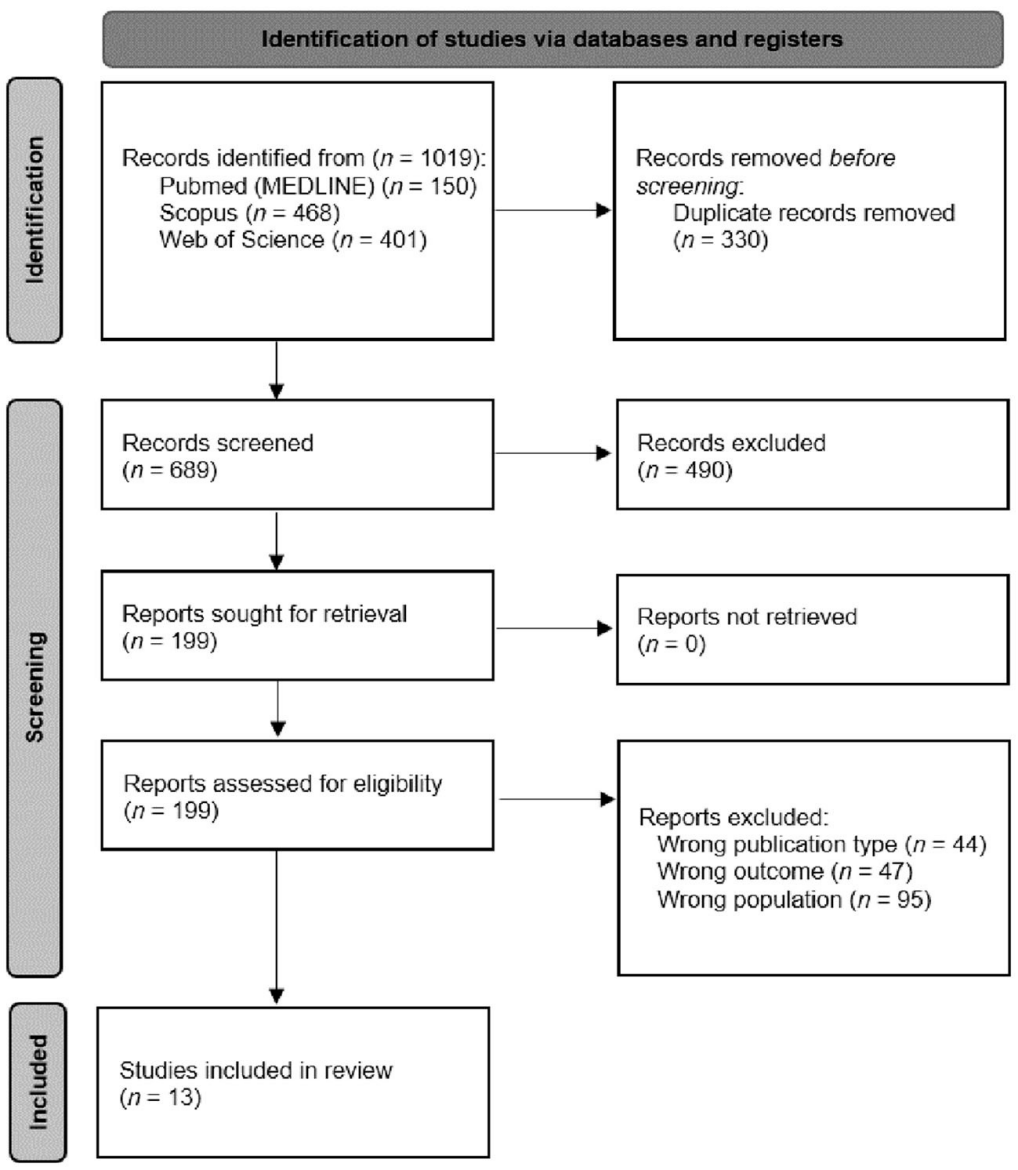
Flow diagram of the study selection process.

The raw search of the databases yielded a total of 1,019 articles, followed by a filter for duplicate articles that left 689 studies to be screened. Subsequently, 490 articles were excluded based on title and abstract review, only 199 full-text articles were reviewed, leaving a total of 13 articles (26–38) that met the inclusion criteria (Figure 1).

### 3.2. Methodological quality assessment

From the thirteen articles included in this study, it was possible to score nine (26–32, 35, 36) of them using the PEDro website, while 4 of the studies (33, 34, 37, 38) were evaluated manually. Two of the studies (29, 35) presented a fair methodological quality while the remaining 11 were between good (26–28, 30, 32–38) and excellent (31). The item that stood out the most was item 6, blinding the therapists, where only 1 of the studies (34) achieved it, additionally, only 4 of the studies (31, 34, 37, 38) blinded the subjects. Table 1 summarizes the evaluation of the methodological quality of the included studies.

**Table 1:** Methodological quality of the articles included.

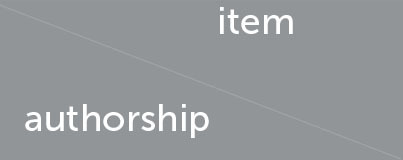	**1**	**2**	**3**	**4**	**5**	**6**	**7**	**8**	**9**	**10**	**11**	**Total**
Nambi et al. (26)	Y	Y	Y	Y	N	N	Y	Y	N	Y	Y	7
Ghasemikaram et al. (27)	Y	Y	Y	Y	N	N	Y	N	Y	Y	Y	7
Kemmler et al. (28)	Y	Y	Y	Y	N	N	Y	Y	Y	Y	Y	8
Bagheri et al. (29)	Y	Y	N	Y	N	N	N	N	N	Y	Y	4
Kemmler et al. (30)	Y	Y	Y	Y	N	N	Y	N	Y	Y	Y	7
Kemmler et al. (31)	Y	Y	Y	Y	Y	N	Y	Y	Y	Y	Y	9
Kemmler et al. (32)	Y	Y	Y	Y	N	N	Y	Y	Y	Y	Y	8
Moghadam et al. (33)	Y	Y	N	Y	N	N	N	Y	Y	Y	Y	6
Nilsson et al. (34)	Y	Y	Y	Y	Y	Y	N	N	Y	Y	Y	8
Mafi et al. (35)	Y	Y	N	Y	N	N	N	Y	N	Y	Y	5
Lichtenberg et al. (36)	Y	Y	Y	Y	N	N	Y	Y	Y	Y	Y	8
Maltais et al. (37)	Y	Y	N	Y	Y	N	Y	N	N	Y	Y	6
Zdzieblik et al. (38)	Y	Y	Y	Y	Y	N	Y	N	N	Y	Y	7

Items: 1: eligibility criteria, 2: random allocation, 3: concealed allocation, 4: baseline comparability, 5: blind subjects, 6: blind therapists, 7: blind assessors, 8: adequate follow-up, 9: intention-to-treat analysis, 10: between-group comparisons, 11: point estimates and variability, Y, Yes; N, No. The eligibility criteria item does not contribute to the total score.

### 3.3. Characteristics of the studies

All articles included in this review were randomized clinical trials. The studies were primarily conducted in Germany (27, 28, 30–32, 35, 36, 38), however, there were also studies from Iran (29, 33), Canada (34, 37) and Saudi Arabia (26). Publication dates ranged from 2015 to 2022, being 2020 the year with the highest number of publications, 6 in total (29–34).

In total, 571 older male adults with sarcopenia or osteosacorpenia participated were selected for inclusion in the studies, with an age range of 63 to 81 years. A total of 291 subjects were assigned to experimental groups that based their intervention on resistance exercise combined with another treatment, 263 participants were assigned to control groups while 17 received an intervention without resistance training. Table 2 presents the characteristics of the included articles.

**Table 2:** Characteristics of the studies.

**Author**	***n*** **IG/CG**	**Age IG/CG**	**Intervention group: type, duration, volume, frequency, and intensity**	**Control group protocol**	**Duration and assessment**	**Outcomes**
Nambi et al. (26)	38/38	63.2 ± 3.1	T: RT + AT	RT + High intensity AT	8 weeks	Handgrip strength; muscle mass; quality of life
		64.1 ± 3.2	V: RT: 3 sets-−10 repetitions/AT: 30 min		T0: baseline	
			F: 4 sessions a week		T1: 4 weeks	
			I: RT: 10 RM/low intensity AT: 40–60% HRmax/high intensity AT: 60–80% HRmax		T2: 8 weeks	
					T3: 6 months	
Ghasemikaram et al. (27)	21/22	79.6 ± 3.6	T: RT + protein, calcium, and vitamins supplementation	Life as usual + protein, calcium, and vitamins supplementatin	18 months	Muscle quality; maximum hip/leg extensor strength per unit of mid-thigh intra-fascia volume and per unit of thigh muscle mass
		80.8 ± 4.7	V: 12–14 exercises/session		T0: baseline	
			F: 2 times per week		T1: 18 months	
			I: high intensity		T2: 24 months	
Kemmler et al. (28)	21/23	77.8 ± 3.6	T: RT + protein, calcium, and vitamins supplementation	Life as usual + protein, calcium, and vitamins supplementatin	6 months	Skeletal muscle mass index; BMD; handgrip strength
		79.2 ± 4.7	V: 12–14 exercises/session		T0: baseline	
			F: 2 times per week		T1: 6 months	
			I: high intensity		T2: 12 months	
					T3: 18 months	
					T4: 6 months after the intervention	
Bagheri et al. (29)	IG1: 10	64.1 ± 3.3	T IG1: RT + ET	Life as usual	8 weeks	1RM estimation on lower limbs; VO2 max
	IG2: 10	63.8 ± 3.6	T IG2: ET + RT		T0: baseline	
	CG: 10	65.0 ± 3.9	V: RT: 6 exercises, 8–16 repetitions/ ET: 15–30 min		T1: 8 weeks	
			F: 3 times a week			
			I: RT: 75% of a RM/ET: 70% HRmax			
Kemmler et al. (30)	21/23	77.8 ± 3.6	T: RT + protein, calcium, and vitamins supplementation	Life as usual + protein, calcium, and vitamins supplementatin	6 months	BMD; STRENGTH OF LOWER Limbs
		79.2 ± 4.7	V: 12–14 exercises/session		T0: baseline	
			F: 2 times per week		T1: 28 weeks	
			I: high intensity		T2: 54 weeks	
					T3: 78 weeks	
Kemmler et al. (31)	21/23	77.8 ± 3.6	T: RT + protein, calcium, and vitamins supplementation	Life as usual + protein, calcium, and vitamins supplementatin	6 months	BMD; prevalence of sarcopenia; handgrip strength; gait velocity
		79.2 ± 4.7	V: 12–14 exercises/session		T0: baseline	
			F: 2 times per week		T1: 28 weeks	
			I: high intensity		T2: 54 weeks	
					T3: 78 weeks	
Kemmler et al. (32)	21/23	77.8 ± 3.6	T: RT + protein, calcium, and vitamins supplementation	Life as usual + protein, calcium, and vitamins supplementatin	1 year	BMD; SMI; maximum dynamic hip- and leg-extensor strength
		79.2 ± 4.7	V: 12–14 exercises/session		T0: baseline	
			F: 2 times per week		T1: 28 weeks	
			I: high intensity		T2: 54 weeks	
					T3: 78 weeks	
Moghadam et al. (33)	IG1: 10	64.1 ± 3.3	T IG1: RT + ET	Life as usual	8 weeks	BMI; 1RM estimation for all the exercises used in the resistance training; satellite cells related markers myogenic factor dietary intake
	IG2: 10	63.8 ± 3.6	T IG2: ET + RT		T0: baseline	
	CG: 10	65.0 ± 3.9	V: RT: 6 exercises, 8–16 repetitions/ET: 15–30 min		T1: 8 weeks	
			F: 3 times a week			
			I: RT: 75% of a RM/ET: 70% HRmax			
Nilsson et al. (34)	16/16	77.4 ± 2.8	T: HBRE + walking + Protein compound	Placebo + HBRE	12 weeks	BMD; hip-waist index; BMI; hear rate; arterial blood pressure; sarcopenia diagnosis; handgrip strength; 1RM on lower limbs; physical performance; muscle quality; physical activity; dietary intake
		74.4 ± 1.3	V: 3 sets/10–15 repetitions		T0: baseline	
			F: 3 sessions a week		T1: 12 weeks	
			I: low intensity HBRT/Walking: 5,000 steps on exercise days and 10,000 steps on rest days			
Mafi et al. (35)	IG1: 14	69.0 ± 2.4	T IG1: RT	Placebo	8 weeks	BMI; dietary intake; maximal muscle strength (leg and chest press); time up and go; physical activity level; follistatin; myostatin
	IG2: 15	69.0 ± 3.1	T IG2: RT + Epicatechin		T0: Baseline	
	IG3: 17	68.6 ± 3.1	T IG3: Epicatechin		T1: 8 weeks	
	CG: 16	68.0 ± 3.0	D: 55 min			
			V: 3 sets/8–12 repetitions/8 exercises			
			F: 3 sessions a week			
			I: 60–80% of 1RM			
			Epicatechin dosage: 1 mg/Kg/day			
Lichtenberg et al. (36)	21/22	77.8 ± 3.6	T: RT + protein, calcium, and vitamins supplementation	Life as usual + protein, calcium, and vitamins supplementatin	6 months	BMD; prevalence of sarcopenia; handgrip strength; gait velocity
		79.2 ± 4.7	V: 12–15 exercises/ 1–2 sets/7–18 repetitions		T0: Baseline	
			F: 2 times per week		T1: 6 months	
			I: High Intensity			
Maltais et al. (37)	IG1: 8	64 ± 4.8	T IG1: RT + EAA supplementation	Chocolate-flavored rice milk placebo	4 months	BMI; muscle mass; maxima muscle strength; physical activity level; dietary intake; walking speed; time up and go; chair stand: standing balance task; one leg stand
	IG2: 8	68 ± 5.6	T IG2: RT + EAAmilk + cowmilk		T0: Baseline	
	CG: 10	64 ± 4.5	D: 60 min		T1: 4 months	
			V: 9 exercises/3 sets/6–8 repetitions			
			F: 3 times a week			
			I: 80% of 1RM			
Zdzieblik et al. (38)	26/27	72.3 ± 3.7	T: RT + protein supplementation	Collagen peptides-based placebo	12 weeks	Body composition; muscular strength; sensory motor control; dietary intake
		72.1 ± 5.5	D: 60 min		T0: Baseline	
			F: 3 times a week		T1: 12 weeks	
			I: High Intensity			

IG, intervention group; CG, control group; T, type; D, duration; F, frequency; I, intensity; HRmax, maximum heart rate; HBRE, home-based resistance exercise; RT, resistance training; ET, endurance training; AT, aerobic training; RM, repetition maximum; BMI, body mass index; BMD, bone mineral density; SMI, structure model index; EAA, essential amino acids.

### 3.4. Characteristics of resistance training-based interventions

Resistance training-based intervention protocols used to treat the subjects differed from study to study. Regarding duration, it varied from 8 weeks (26, 29, 33, 35) to 18 months (27), including interventions of 12 weeks (34, 38), 4 (37), 6 (30–32, 36), and 12 months (32). 8 studies (27–33, 36) determined volume based on the number of exercises and repetitions while 2 studies (26, 34) established it using sets and repetitions, 2 studies (35, 37) reported volume combining both forms in addition to quantifying the duration of the session and 1 study (38) established the volume from the duration of the session only. It is important to highlight that 6 of the studies analyzed (27, 28, 30–32, 36) followed exactly the same protocol, which was denominated The Franconian Osteopenia and Sarcopenia Trial (FrOST).

Only 2 studies (34, 35) included an intervention group that performed resistance training without any complementary intervention or used placebos, finding better results when they combined their resistance training protocol with some other intervention.

### 3.5. Effects of resistance training combined with protein and vitamin supplementation

Six of the studies (27, 28, 30–32, 36) analyzed in this review included as complementary treatment the use of protein and vitamin supplements; in addition, all employed the same protocol of high-intensity resistance training for IG compared with physical inactivity in CG. These similarities in the intervention protocols are due to the fact that all 6 articles are part of the FrOST.

Regarding protein and vitamin supplementation, it consisted of supplying all participants (GC and GI) with dietary protein, the GI was given 1.5 g/kg/day while the CG only received 1.2 g/kg/day, additionally, both groups received 800 IE/day of vitamin supplements. However, although all the studies are part of the FrOST, each one observed different variables and at different times, finding positive effects on the Skeletal Muscle Index (SMI) (28, 31, 32, 36), Bone Mineral Density at the lumbar and hip region (28, 31, 32), maximal hip and leg extensor strength (27, 28, 30, 32), handgrip strength (28, 31, 36), fat-free mass and abdominal and total body fat measured by DXA (30), prevalence of sarcopenia measured through z-score (31, 36), Gait velocity (31, 36) and, finally, muscle quality (27).

Two articles (27, 28), besides evaluating the pre and post intervention changes, conducted a 6 months follow-up of the patients to assess the effects that the detraining process has on this population. Kemmler et al. observed that 6 months after the end of the intervention a statistically significant and greater worsening was presented in the IG than in CG of SMI and hip extensor strength, which is partially consistent with the findings of Ghasemikaram et al. (27), who observed a significant worsening in maximal hip and leg extensor strength, however, when comparing both groups, the IG results were still better and presented statistically significant differences vs. CG.

### 3.6. Effects of resistance training combined with protein only supplements

Four of the studies analyzed (34, 35, 37, 38) used specific supplementations based on different proteins in addition to resistance training. Nilsson et al. (34) used a protein compound called Muscle5 combined with a RT protocol based on low intensity exercises resisted by elastic bands and compared it with the same RT protocol but combined with a placebo, finding that when RT is combined with Muscle5 it generates better results in terms of fat-free mass, grip strength as well as press RM, functionality, muscle cross-sectional area and total lean mass. Mafi et al. (35) combined RT with Epicatechin, a flavonoid recognized for its ability to generate muscular mitochondria, reporting favorable effects on follistatin levels and maximal strength measured in chest and leg press; these effects were statistically significant when compared with the RT-only group and the CG (placebo). On the other hand, Maltais et al. (37), used as a complementary intervention to RT in the IG1 an Essential Amino Acids (EAA) supplement while for IG2 they used EAA from milk and compared them with a placebo based on flavored rice milk, finding that the RT groups had favorable results on the variables analyzed, however, there were no significant differences between the two IG which allowed them to conclude that RT is favorable for patients with sarcopenia regardless of the type of protein supplementation used, however, it is still important. Finally, Zdzieblik et al. used Collagen Peptides as an adjunctive intervention and observed significantly more pronounced changes in the IG in relation to fat-free mass, isokinetic quadriceps strength and fat mass, demonstrating that the combination of RT with Collagen Peptides is ideal for improving body composition in men with sarcopenia.

### 3.7. Effects of resistance training combined with aerobic exercise

Three of the studies (26, 29, 33) evaluated the effects of resistance training combined with aerobic training (AT). On the one hand, Bagheri et al. (29) and Moghadam et al. (33) studied the effects of these interventions by combining them in different ways in two IG. IG1 performed RT and then AT while in the IG2 this order was inverted, besides this, the other variables around the training prescription remained the same. On the other hand, Nambi et al. (26) only studied the combination of RT followed by AT.

The AT included a variety of exercises, two of the studies (29, 33) performed exercise on a fixed-speed cycle ergometer at an intensity of 70% for 30 min, while Nambi et al. (26) based their protocol on 30 min of aerobic activity comprising 20 min on the treadmill and 10 min on cycle ergometer, in this study they used aerobic exercise in the two groups analyzed, group 1 performed AT at low intensity (40–60% HRmax) while group 2 performed high intensity AT (60–80% HRmax).

Among the variables considered by the studies, it was found that resistance training combined with low-intensity aerobic training improved handgrip strength (26), quality of life (26), muscle mass (26, 29), musculoskeletal mass (33), total fat percentage (29), weight (29), el BMI (29), lower and upper limb power (29, 33), VO2Max (29, 33) and generated favorable modifications in the Satellite Cells related markers (33). Additionally, it was observed that low-intensity aerobic training is more effective than high-intensity training when combined with resistance training (26), irrespective of AT and RT order (29, 33).

## 4. Discussion

The objective of this systematic review was to determine the effects of resistance training when combined with different complementary interventions on musculoskeletal health in older male adults with sarcopenia. For this purpose, 13 articles were reviewed that included resistance exercise as the main treatment and employed other types of complementary treatments to enhance its effects. This review revealed robust evidence on the effects of resistance training when combined, mainly with aerobic exercise or with different nutritional supplementation, on variables associated with musculoskeletal health.

Regarding the quality of the included studies, it is possible to determine that the quality did not influence the observed results, since only one of the articles had a quality below Good and the results were homogeneous. Additionally, it is important to highlight that only one of the articles performed a blinded intervention, principally due to the fact that blinding an exercise-based intervention is a common problem reported in multiple reviews and studies (39, 40).

Resistance training is a widely used intervention for the treatment of patients with sarcopenia, due to the diverse effects it has on musculoskeletal health (16, 41, 42). However, due to the increasing number of people who suffer from this disease, it is necessary to develop interventions to obtain more and better results that also last over time, therefore, the effects of resistance exercise have been studied in combination with other types of intervention.

The implementation of protein supplementation either combined with vitamins (27, 28, 30–32, 36) or alone (34, 37, 38) along with RT showed favorable effects on multiple musculoskeletal health variables, mainly those associated with strength and body composition. These results may be due to the well-known important role of amino acids as substrates of protein synthesis and as mediators in physiological and metabolic processes (43). One of the conditioning factors of the effects of this type of supplementation is the origin of the protein, Poortmans et al. (44) that when RT is performed and accompanied by a whey protein-based supplement, the absorption process and its effects on protein synthesis are better than when other sources of EAA are used. However, although the studies analyzed did not all employ whey protein-based supplements (37), they all evidenced favorable results on the variables evaluated. Additionally, it is important to consider the negative effects of high protein intake on liver and colon health (43), therefore the doses prescribed to subjects should be correctly calculated.

Another complementary intervention to RT was AT, which has been shown to be better at modulating the immune system and inflammatory markers in older adults than other types of exercise (45). The results obtained in the studies analyzed in this review are congruent with those of several authors (46–48), who applied a similar protocol of RT combined with AT in prefrail or frail older adults, finding that it increased strength, muscle power and aerobic capacity. Although the order of the interventions was not a determinant of treatment success as demonstrated by Bagheri et al. (29) and Moghadam et al. (33), the intensity of the AT was. According to the findings of Nambi et al. (26), low-intensity AT was better than high-intensity AT when combined with RT, this is because during low-intensity exercise the oxidative capacity of the cell is stimulated and blood flow is increased (49), leading to significant differences in multiple variables, including strength, possibly due to the differences in energy metabolism that low-intensity AT produces.

With regard to the detraining process, two articles (27, 28) observed that after 6 months following the intervention, the effects obtained partially disappeared, decreasing about a third of what was gained, which agrees with the results of Padilha et al. (50), who after a 12-week intervention based on RT in older women observed that, although detraining negatively affects this population, it is not enough to reverse all the changes resulting from exercise. This can be attributed to the principle of stability of adaptation, which states that the longer the period of training time, the more stability it will create and the less susceptibility to failure it will present. Another possible explanation is related with the principle of reversibility of training, which establishes that once the exercise is interrupted, the benefits obtained and the adaptations generated are progressively lost; however, there is no specific time for this. The reversibility process depends on several factors such as: the duration of the training period, how significantly the subject's behavior changed, as well as intrinsic characteristics of the subject, such as age and genetics (51).

Likewise, it seems that the intensity of RT has no impact on its effects, since all studies obtained favorable results whether they used high (27, 28, 30–32, 36), moderate (26, 29, 33, 35, 37, 38) or low intensity (34). These results are partially in agreement with those of Steib et al. (52), which in their systematic review with meta-analysis determined that high-intensity RT is better than low-intensity RT in relation to strength; however, in terms of functionality there is no difference. Multiple studies have evidenced an association between strength and functionality (53, 54), which means that improvements in strength after a certain point do not necessarily mean improvements in functionality (55), therefore, different RT intensities could have similar effects on this variable.

Finally, this review presents several limitations that should be considered when interpreting the results. On the one hand, the great variety in the prescription of RT does not allow us to establish an ideal treatment protocol for older male adults with sarcopenia. In addition, due to the fact that 84.6% of the articles were published in Eurasia and, specifically, more than 60% of the articles were published in Germany, the results cannot be extrapolated to other populations such as Latin American or African populations. Also, because of the variety in the methodological quality of the articles and the lack of a specific PEDro score inclusion criteria, the quality of this systematic review may be affected.

## 5. Conclusion

Interventions based on RT have beneficial effects on different variables associated with musculoskeletal health in older male adults with sarcopenia. RT training can be used at any intensity as long as the objective is to improve functionality; additionally, when combined with AT, the AT should be low intensity to optimize results. Nutritional supplementation enhances the effects of RT, but by itself is not sufficient treatment for this population, and doses should be carefully adjusted to avoid potential health problems in the future. RT must be constant for maintaining the results obtained. Finally, RT is a cost-effective, low-risk strategy for treating sarcopenia, and is always recommended to be combined with another type of intervention, either aerobic exercise or nutritional supplementation to enhanced the effects.

## Data availability statement

The original contributions presented in the study are included in the article/supplementary material, further inquiries can be directed to the corresponding author.

## Author contributions

Conceptualization: MC-F and ML-N. Methodology: AA-A and FH-C. Performing literature review and synthesis of literature: MC-F, AA-A, and DA-R. Quality assessment: CR-L, FH-C, and AA-O. Writing—original draft preparation: MC-F, AA-A, and YC-C. Writing—reviewing and editing: FH-C, AA-O, YC-C, and DA-R. All authors have read and agreed to the published version of the manuscript.
